# Insulin-Like Growth Factor-1 (IGF-1) Reduces ischemic changes and increases circulating angiogenic factors in experimentally - induced myocardial infarction in rats

**DOI:** 10.1186/2045-824X-3-13

**Published:** 2011-06-09

**Authors:** Mathews Lisa, Nagaraja Haleagrahara, Srikumar Chakravarthi

**Affiliations:** 1 Division of Human Biology, Faculty of Medicine, International Medical University, Kuala Lumpur, 57000, Malaysia; 2 Division of Pathology, International Medical University, Kuala Lumpur, 57000, Malaysia

## Abstract

**Background:**

Coronary artery disease is a global health concern in the present day with limited therapies. Extensive efforts have been devoted to find molecular therapies to enhance perfusion and function of the ischemic myocardium. Aim of the present study was to look into the effects of insulin like growth factor -1 (IGF-1) on circulating angiogenic factors after myocardial ischemia in rats.

**Methods:**

Adult male Sprague-Dawley rats were randomly divided into 10-days control, myocardial infarction, IGF-1 alone (2 μg/rat/day) and ISO+IGF-1 groups. Isoproterenol (ISO), a synthetic catecholamine was used to induce myocardial infarction. Serum transforming growth factor-β (TGF-β) and vascular endothelial growth factor (VEGF) levels were checked after 10-days of IGF-1 administration.

**Results:**

There was a significant increase in heart weight after IGF-1 treatment. A significant increase in cardiac enzyme level (CK-MB and LDH) was seen in isoproterenol treated rats when compared to control group. IGF-1treatment induced a significant increase in serum angiogenic factors, IGF-1, VEGF and TGF beta levels. IGF-1 also reduced the ischemic changes in the myocardium when compared to the isoproterenol alone treated group.

**Conclusions:**

In conclusion, treatment with insulin-like growth factor-1 (IGF-1) in myocardial infarction significantly increased circulating angiogenic growth factors like IGF-1, VEGF and TGF beta thus, protecting against myocardial ischemia.

## Background

Cardiovascular disease is the leading cause of mortality, not only in the Western world, but also in developing countries [1]. Coronary artery disease is a global health concern today, with limited treatment options available to address this disorder. Extensive efforts have been devoted to molecular therapies to enhance perfusion and function of the ischemic myocardium [2]. Angiogenesis is a process involving the formation of new blood vessels from the pre-existing vasculature that occurs in many physiological and pathological situations [3]. Although neovascularization is an important vascular response to chronic hypoxia, the role of angiogenesis in myocardial ischemia remains unclear [4,5]. Therapeutic angiogenesis is being tested as a novel treatment for ischemic heart disease [2]. In this connection, the challenge in the last decennium has been to find methods of inducing new vascular growth in the ischemic myocardium of patients suffering from atherosclerotic coronary artery disease where treatment by balloon angioplasty or coronary by-pass grafting is inappropriate. Therapeutic angiogenesis with recombinant vascular endothelial growth factor proteins or with the genes encoding the proteins holds new promise for the treatment of cardiovascular disease [6-9].

Ischemic changes are known to induce a significant alteration in the levels of circulating angiogenic and anti-angiogenic factors. Members of the family of vascular endothelial growth factors (VEGFs) are major stimulators of the growth of new blood vessels. Among the most potent growth factors that induce angiogenesis, besides the VEGFs, are the fibroblast growth factors. Together with vascular endothelial growth factors and hepatic growth factors, they are known to have a revascularization stimulating effect after ischemia [10,11].

Insulin-like growth factor-1 (IGF-1) is a peptide hormone structurally related to insulin, and which has a pleiotropic effect on cell growth and metabolism. IGF-1 is among several factors that have been suggested to regulate predegenerative abnormalities [12]. The biological actions attributed to IGF-1 include its chemo-attractant properties, ability to release cytokines, promotion of angiogenesis and stimulation of extracellular matrix production [13]. There are no reports on the effect of exogenous IGF-1 on angiogenesis in the ischemic heart. We hypothesized that IGF-1 could play a protective role in myocardial ischemia by enhancing the circulating levels of angiogenic factors.

Myocardial injury induced by isoproterenol, a synthetic catecholamine, is a standard experimental model for the investigation of pharmacological protective effects against ischemic injury [14]. The mechanism of isoproterenol-induced myocardial injury includes cytosolic calcium overload, lipid peroxide generation and pro-coagulant activity [15,16]. The pathological process of isoproterenol-induced myocardial damage is characterized by patchy areas of myocardial ischemia. This myocardial injury has many similarities to the traditional myocardial infarction attributed to ischemic coronary disease [17].

The aim of the present research was to determine whether a short term administration of IGF-1 had an effect on circulating angiogenic factors following myocardial ischemia in rats.

## Methods

### Animals

Adult male Sprague-Dawley rats (250-300 g) were acclimatized over a week to standard laboratory conditions (24 ± 3°C ambient temperature, 40-60% relative humidity, and 12 h light-dark photoperiods) with food and water *ad libitum*. Each rat was individually housed with fresh bedding, food and water reservoir at the commencement of the treatments. Protocols for the use of animals and all the experimental procedures were approved by the Institutional Research and Ethics Committee.

### Experimental treatments and assays

The rats were randomly assigned to the following groups (six rats in each group): (a) Control (b) Isoproterenol-induced myocardial ischemia; (c) Insulin-like growth factor-1 (IGF-1) alone and (d) Isoproterenol-induced myocardial ischemia followed by treatment with IGF-1. Isoproterenol (Sigma-Aldrich, USA; 85 mg/kg body weight) was used to induce myocardial ischemia in rats by subcutaneous injection for two consecutive days. For treatments with IGF-1 (Sigma Aldrich, USA), 2 μg/rat/day were subcutaneously administered daily for 10-days. Control rats were injected with saline.

Twenty four hours after the last treatment, the rats were anaesthetized with ether, and blood samples were collected via cardiac puncture. The blood samples were centrifuged at 6000 rpm at 4°C for 15 minutes to separate out the serum that was stored at -80°C for biochemical analysis.

The heart was dissected out, weighed, and a portion of it was preserved in 10% formalin. Paraffin blocks were prepared from the heart samples and thin sections (4 μM) were prepared and stained with haematoxylin-eosin (H&E) for light microscopy. The sections were examined for necrosis, nuclear pyknosis, hypertrophy, angiogenesis, scar formation and macrophage activity. Commercially available enzyme-linked immunosorbant assay (ELISA) kits were used for the quantitative determination of creatinine kinase-MB (CK-MB; *BioCheck, Inc., USA*), insulin-like growth factor-1 (IGF-1; *USCN Life, China*), vascular endothelial growth factor (VEGF; *USCN Life, China*) and transforming growth factor-beta (TGF-ß *BioVendor, Czech Republic*) concentrations in rat serum.

### Creatinine kinase - MB Assay

The assay system utilized a primary monoclonal antibody directed against an antigenic determinant on the CK-MB. The antibody-enzyme conjugate consisted of goat anti-CK-MB seconday antibody conjugated to horseradish peroxidase (HRP). Colour development of the TMB (3,3'5,5' tetramethyl-benzidine) chromogenic substrate was measured by absorbance at 450 nm.

### Insulin-like Growth Factor (IGF-1) Assay

The micro titre plate provided in this kit was pre-coated with a primary antibody specific to IGF-1. Standards or samples are then added to the appropriate micro titre plate wells together with a biotin-conjugated seconday polyclonal antibody specific for IGF-1. Avidin conjugated to horseradish peroxidase (HRP) and a TMB (3,3'5,5' tetramethyl-benzidine) substrate completed the immuno-reaction sequence that was ended with the addition of a sulphuric acid solution. Colour change was measured at 450 nm.

### Transforming Growth Factor-β (TGF-β) Assay

In this assay, rat TGF-β present in samples bound to the adsorbed primary anti-rat TGF-β antibodies in the micro titre wells. A HRP-conjugated secondary monoclonal anti-rat TGF-β antibody bound to rat TGF-β captured by the primary antibody. The reaction between HRP and substrate solution was stopped by the addition of an acidic solution and colour change was measured at 450 nm.

### Vascular Endothelial Growth Factor (VEGF) Assay

The micro titre plate provided in this kit was pre-coated with the primary antibody. Standards or samples are added to the appropriate micro titre plate wells together with the seconday biotin-conjugated polyclonal antibody preparation specific for VEGF. Avidin conjugated to horseradish peroxidase (HRP) was added, followed by the TMB (3,3'5,5' tetramethyl-benzidine) chromogenic substrate solution. The reaction was stopped by the addition of acid and absorbance was measured at 450 nm.

Lactate dehydrogenase (LDH) activity was determined using a commercial kit (LDH; *BioAssay Systems, USA*). In this reaction, the enzyme substrate was oxidized with the transfer of electrons to the tetrazolium salt MTT. Absorbance of the reduced MTT was read at 565 nm and LDH activity was calculated according to the equation provided in the kit.

### Statistical Analysis

Global comparison among treatments was carried out using the Kruskal Wallis non-parametric test, and pair-wise comparisons between different groups were performed using the Mann-Whitney-U test. The significance of difference between treatments was set at p < 0.05.

## Results

**Figure 1:**
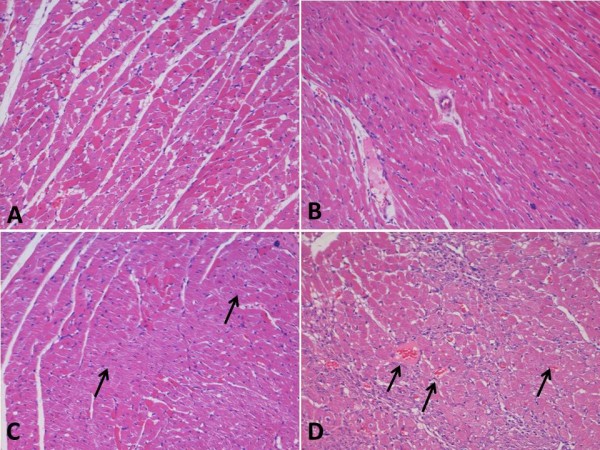
**Histopathological analysis of rat myocardium:** A - Normal appearance myocardial tissue in control rats. B - Normal myocardium without any necrosis or nuclear pyknosis in IGF-1 alone group, C - Myocardial infarction with ISO treatment (arrows) showing necrosis and scar formation, D - Vascular proliferation and macrophage activity in ISO + IGF-1 treated group. There is a local angiogenic reaction in the myocardium with many small vessels in the surrounding area (arrows), ×100.

Histophathological examination showed robust myocardium architecture and normal morphology in the control group of rats. On the other hand, the isoproterenol group displayed evidence of myocyte necrosis, neutrophilic infiltration and areas of diffuse interstitial oedema, suggestive of myocardial infarction. Rats treated with insulin-like growth factor alone had normal myocardium morphology with mild vascular proliferation whereas those administered isoproterenol (ISO) showed evidence of myocyte necrosis. This development was observed also in rats treated with both isoproterenol (ISO) and IGF-1, but to a lesser degree when compared with ISO treatment alone. IGF-1 treatment induced capillary sprouting in the necrotic areas, with more prominent macrophage activity and scar formation, suggestive of healing. The wound healing shows infilltration by inflammatory cells and fibrosis, and most importantly there is a local angiogenic reaction in the myocardium with many small vessels in the surrounding area (Figure 1).

There was a significant (p < 0.05) decrease in mean body weight of rats 10 days after ISO injection, but such a change was not observed after IGF-1 administration to the ISO-treated rats. IGF-1 alone increased the mean body weight, but this was not statistically significant. An increase in mean heart weight (p < 0.05) was recorded after 10 days following IGF-1 treatment alone, as compared with controls. Myocardial ischemia arising from ISO treatment significantly increased (p < 0.05) heart weight of rats when compared with corresponding measurements in both the treated only with IGF-1 and control rats. IGF-1 applied to rats injected with ISO attenuated the increase in heart weight due to ISO (p < 0.05; Table 1).

**Table 1:** Effect of insulin-like growth factor-1 on body weight and heart weight

Parameters	Control	IGF-1	M I	M I + IGF-1
Body weight (g)	275.7 ± 4.18	282.18 ± 8.15	268.88 ± 2.17 *	270.44 ± 4.12

Heart weight (g/100 g b.wt)	0.258 ± 0.017	0.287 ± 0.012*	0.321 ± 0.019 * ^✞^	0.310 ± 0.014 * ^✞ ■^

Values are Mean ± SEM.

* Indicates significant difference when compared to control group (P < 0.05)

^✞ ^Indicates significant difference when compared to IGF-1 group (P < 0.05)

^■ ^Indicates significant difference when compared to M I group (P < 0.05)

Compared with the untreated control rats, there were significant increases (p < 0.05) in the cardiac enzymes, CK-MB and LDH, 10 days after the induction of myocardial ischemia by ISO treatment. However, these differences were not observed between the control rats and rats that were administered IGF-1 alone. Treatment with IGF-1 in myocardial ischemia rats significantly reduced (p < 0.05) CK-MB and LDH levels when compared with untreated myocardial ischemia rats. Nevertheless, the levels of cardiac enzymes were still significantly elevated in rats treated with ISO and IGF-1, as compared with the control rats or rats injected with IGF-1 alone (p < 0.05) (Table 2).

**Table 2:** Effect of insulin-like growth factor-1 on cardiac biomarkers

Parameters	Control	IGF-1	M I	M I + IGF-1
CK-MB (ng/mL)	3.319 ± 0.773	3.631 ± 0.669	5.662 ± 0.249 *^✞^	4.262 ± 0.289 * ^✞ ■^

Lactate Dehydrogenase (IU/L)	24.574 ± 2.748	25.403 ± 2.664	80.040 ± 5.774 * ^✞^	59.003 ± 2.688 *^✞ ■^

Values are Mean ± SEM.

* Indicates significant difference when compared to control group (P < 0.05)

^✞ ^Indicates significant difference when compared to IGF-1 group (P < 0.05)

^■ ^Indicates significant difference when compared to M I group (P < 0.05)

When serum IGF-1 levels were compared among the different experimental groups, we observed that there was a significant increase (p < 0.05), compared with control, in IGF-1 level ten days after commencement of the ISO treatment that led to myocardial ischemia. Serum IGF-1 levels also showed a significant increase (p < 0.05) in rats following treatment with IGF-1 alone and even more so in rats treated with both IGF-1 and ISO (p < 0.05; Table 3).

**Table 3:** Effect of insulin-like growth factor - 1 circulating angiogenic factors

Parameters	Control	IGF-1	M I	M I + IGF-1
IGF-1 (ng/mL)	3.884 ± 0.645	8.374 ± 0.552	5.668 ± 0.592 * ^✞^	9.086 ± 0.877 * ^✞ ■^

TGF-β (ng/mL)	6.419 ± 0.878	7.984 ± 0.429	7.763 ± 0.707 *	10.400 ± 0.206 * ^✞ ■^

VEGF (pg/mL)	146.5 ± 9.840	185.0 ± 7.291	224.5 ± 4.832 * ^✞^	318.6 ± 9.661 * ^✞ ■^

Values are Mean ± SEM.

* Indicates significant difference when compared to control group (P < 0.05)

^✞ ^Indicates significant difference when compared to IGF-1 group (P < 0.05)

^■ ^Indicates significant difference when compared to M I group (P < 0.05)

Pair-wise comparisons showed that the angiogenic factors TGF-β and VEGF levels associated with the ISO treatment alone, and ISO with IGF-1 treatment increased significantly (p < 0.05) when compared with the control level (Table 3).

## Discussion

Our study showed that administration of isoproterenol (ISO) (85 mg/kg) to rats for two consecutive days resulted in severe myocardial injury, which was confirmed by elevations in heart weight and in levels of serum CK-MB and LDH. This finding is in accordance with previous studies that used ISO-induced myocardial injury as an experimental model for the investigation of pharmacological protective effects of several drugs against ischemic injury [18]. The ISO treatment produced functional alterations to the myocytes, as manifested by an increase in both the heart weight and serum levels of the biomarkers for cardiac injury, CK-MB and LDH. The biochemical results described in this study are in consonance with earlier reports on ISO-induced myocardial injury where increased cardiac biomarker levels were observed [16]. Panda and Naik [19] in their research study reported that there was a significant alteration in biochemical parameters (increased levels of AST LDH and CK-MB in serum) with the induction of myocardial necrosis using ISO. Zhang et al. showed that a high-dose of ISO produced relative hypoxia leading to ischemia by excessive activation of β_1_-adrenergic receptors that in turn resulted in increased inotropic, chronotropic and dromotropic effects [20]. Another mechanism that might operate is through the formation of reactive oxygen species (ROS) arising from the auto-oxidation of ISO leading to peroxidative damage [21].

In our study, we found that circulating levels of angiogenic factors (TGF-β and VEGF) and the proangiogenic growth factor IGF-1 were significantly increased after ISO treatment. Ischemic injury is known to cause the release of angiogenic factors such as cytokines and vascular growth factors derived from the local tissue area into the blood circulation (6, 7, 8). Our findings showed that ischemic injury caused by ISO induced increased secretion of the angiogenic factors. This could be an adaptive response of the myocardium for repair and revascularization to maintain oxygen supply after myocardial hypoxia [22]. As reported by Folkman [23], physiological response to the development of tissue ischemia included the up-regulation of angiogenic growth factors and mobilization of circulating cellular elements that together enabled development of an accessory vasculature. Rabinovsky et al. [13] opined that angiogenesis occurred after ischemia when stimulated by circulating angiogenic factors that helped in the regulation of endothelial cell migration, cell survival and endothelial cell proliferation [8,9,11,13].

There are numerous other known angiogenic factors apart from the ones studied in this research that are known to play an important role in revascularization after ischemia in various tissues in the body. These include angiogenin, angiopoietin-1, hepatocyte growth factor and platelet derived growth factor [24]. There have been no reports so far on the effect of circulating angiogenic factors on myocardial infarction, except for the role of VEGF on ischemic-induced changes in angiogenesis that have been studied extensively. VEGF is known to induce angiogenesis in the ischemic myocardium by stimulating endothelial cell proliferation and migration [25]. Ma et al. found in their study that TGF-β also played a role in angiogenesis by stimulating extracellular matrix production, stabilizing newly formed vessels and influencing the expression of other angiogenic factors [26].

From our present study, significant increases in circulating levels of VEGF and TGF-β were observed after insulin-like growth factor-1 (IGF-) treatment at a dose of 2 μg/rat/day in rats. Insulin-like growth factor is a potent angiogenic agent [8,9,27-29] that promotes angiogenesis in several physiological and pathological conditions. *Boucher et al*. [30] showed that IGF-1 had proangiogenic effects even at a dose as low as 1 μg/kg. IGF-1 is postulated to induce angiogenesis through interaction with locally produced factors such as VEGF [28,31]. IGF-1 may increase the release of other endothelial cell-derived angiogenic factors into the circulation when this factor binds to its receptors on endothelial cells [32]. Our findings confirmed that one of the mechanisms by which IGF-1 promoted angiogenesis might be by stimulating the release of other angiogenic factors such as VEGF and TGF-ß, which were elevated in the serum of rats treated with IGF-1. These responses in VEGF and TGF-ß levels were further elevated in rats treated with both IGF-1 and ISO, the latter inducing myocardial ischemia. Overall, histopathological comparison of hearts from rats in ischemic group and IGF-1treated groups showed that an obvious angiogenic reaction occurred as a result of IGF treatment. Neovascularization by such angiogenic agents may explain the cardioprotective action of IGF during myocardial ischemia, the mechanism of which has yet to be fully elucidated [33].

Another finding in the present study was that the levels of both the biomarkers of cardiac injury, CK-MB and LDH, were significantly attenuated after IGF-1 treatment of rats with induced myocardial ischemia. These are positive findings suggesting that IGF-1 has a beneficial cardioprotective role in ischemic heart disease.

## Conclusions

IGF-1 treatment enhanced circulating levels of angiogenic factors TGF-β and VEGF, while it reduced the enzymes associated with cardiac injury (CK-MB and LDH) in rats with induced myocardial infarction. IGF-1 treatment may be useful in enhancing regional myocardial blood flow in the injured myocardium via the stimulation of neovascularization attributable to the increased presence of angiogenic factors. IGF-1 acts as a critical permissive agent for the action of other angiogenic growth factors in vascularisation in the myocardium under ischemic conditions. Thus, IGF-1 has the potential to be a novel and efficient therapeutic strategy for myocardial infarction in humans for enhancing angiogenesis.

## Competing interests

The authors declare that they have no competing interests.

## Authors' contributions

NH conceived and coordinated the study, as well as finalized the preparation of the manuscript. LM carried out the experimental work, performed the data analysis and prepared the results. SC carried out the histopathological analysis. All authors read and approved the final manuscript.
